# Fragile X Syndrome: Recent Research Updates toward Capturing Treatments’ Improvement in Clinical Trials

**DOI:** 10.3390/brainsci12101276

**Published:** 2022-09-22

**Authors:** Dejan B. Budimirovic, Dragana D. Protic

**Affiliations:** 1 Department of Psychiatry, Fragile X Clinic, Kennedy Krieger Institute, Baltimore, MD 21205, USA; 2 Department of Psychiatry & Behavioral Sciences-Child Psychiatry, Johns Hopkins University School of Medicine, Baltimore, MD 21205, USA; 3 School of Medicine, University of Belgrade, 11000 Belgrade, Serbia

This *Brain Sciences 2020* Special Issue of nine manuscripts contribute novel data on treatment updates in fragile X syndrome (FXS). The Updated Report on tools to measure outcomes of clinical trials in FXS published in 2017 by an expanded 2013 Working Groups found that most outcome measures had limited information on its psychometric properties and lacked objective measures, particularly in the behavioral domain, which indicated that endpoint development in FXS needed to continue with an emphasis on objective measures [1]. The data provided here address the lack of objective data (directly observable or/and direct testing, quantifiable, biomarkers) to fill the gap in the endpoints and perhaps of the brain function in FXS, including to capture meaningful improvements in quality of life of individuals and families with FXS [1,2]. Specifically, the articles that contributed to that effort included here are on: (1) a comprehensive *FMR1* genotype-protein-FXS phenotype profiles evaluation (Budimirovic et al.); (2) psychometric properties of an adaptive functioning measure (Cordeiro et al.); (3) delineating repetitive behavior profiles in FXS with and without ASD across the lifespan (Reisinger et al.); (4) a potential direct measure of social interaction skills in individuals with FXS (Cordeiro et al.); (5) validity of existing standardized and new promising sensitive to treatment of language-related assessments in FXS (two articles: 5a Shaffer et al. and 5b Hoffmann et al.); (6) correlates to health-related quality of life in FXS (Coffman et al.); (7) a meta-analysis of the response to placebo in FXS controlled trials (Luu et al.); and (8) a further analysis of a computer-based cognitive Cogmed FXS controlled trial data (Scott et al.).

Fragile X syndrome is the most common inherited cause of intellectual disability (ID) and the most common known single-gene cause of ASD [3,4]. A CGG trinucleotide-repeat expansion full mutation (FM, >200 CGG repeats) in the promoter region of the Fragile X messenger ribonucleoprotein 1 gene (*FMR1*) leads to the hypermethylation epigenetic silencing of the gene, which subsequently results in the reduction in or absence of Fragile X Messenger Ribonucleoprotein (FMRP) [5,6]. FMRP, a multifunctional RNA-binding protein, is critical for synaptic function in the brain and interacts with approximately 4% of the total mRNAs in the mammalian brain [5,6]. Therefore, marked reduction in the expression of *FMR1* transcript and FMRP due to a fully or mostly methylated *FMRI* gene nearly always results in global neurodevelopmental delay, clinically termed FXS, especially in males [5].

(1). Budimirovic and colleagues conducted a comprehensive genotype–phenotype evaluation of *FMR1*/FMRP profiles in FXS and their associations with the FXS neurobehavioral phenotype. Understanding the relationship between the *FMR1* DNA–RNA–protein pathway and neurobehavioral phenotype is a top priority within this prototypical genetic disorder and has the potential to provide far-reaching insight into other less-common neurogenetic conditions associated with ID and ASD. In this study, the authors compared nine sensitive and quantitative assays for assessing *FMR1* DNA, RNA, and protein using a reference set of well-characterized *FMR1* cell lines. The most informative assays were them applied to examine molecular–neurobehavioral relationships in an independent cohort of predominantly pediatric and male patients with FXS with and without ASD. In addition to these primary analyses, the authors contrasted molecular profiles in matched blood and buccal specimens to determine their value in informing FXS phenotypical variability [5]. Results from this study including *FMR1* CGG repeat expansion, methylation levels, and FMRP levels in both cell lines and blood samples were consistent with findings from previous *FMR1* genomic and protein studies and further supported previous reports demonstrating relatively high prevalence of methylation mosaicism. Molecular–neurobehavioral correlations confirmed the inverse relationship between overall severity of the FXS phenotype and decrease in FMRP levels, in addition to revealing a significant association between a diagnosis of FXS with ASD and two-fold lower levels of FMRP, observed in younger-age and IQ-adjusted males, compared to FXS without ASD. Those with severe ID had even lower FMRP levels independent of ASD status in the male-only subset. Importantly, these results underscore the link between *FMR1* expansion, gene methylation, and FMRP deficit and unveiled preliminary evidence supporting the relationship between ASD and other neurobehavioral features and the magnitude of FMRP deficit [5]. Given the importance of these discoveries, this article has been chosen as an ‘Editor’s Choice’ article based on recommendations by the scientific editors of MDPI journals.

(2). A study by Cordeiro and colleagues evaluated some of the psychometric qualities of the Pediatric Evaluation of Disability Inventory Computer Adaptive Test (PEDI-CAT) in FXS. They focused on the revised version of the adaptive functioning measure and used the Vineland Adaptive Behavior Scales-Third Edition (VABS-3 as a comparable measure. While VABS-3 is consistently utilized as a gold standard adaptive functioning measure in large-scale national research efforts, it can be lengthy to administer by interview, the instructions may not be clear to many parents/caretakers, and the scores may often be inconsistent compared to those obtained through the interview approach. Thus, the authors evaluated the feasibility, validity, and test–retest performance of the PEDI-CAT in FXS. This study revealed that males and females did not significantly differ on the test domains, except for daily activities, and the test showed more floor effects in the mobility and social-cognitive domains in comparation with VABS-3. The test’s daily activities, mobility, social-cognitive, and responsibility domains were all significantly correlated with most of the VABS-3 domains. The results of this study suggested that the PEDI-CAT is efficient and minimal training is needed to administer it. However, it lacks specificity and shares a high rate of floor effects with the VABS-3 [7].

(3). Using longitudinal data derived from one of the largest available Fragile X clinical registry, Reisinger and colleagues examined the restricted and repetitive behaviors (RRB) profile of males and females with FXS across the lifespan. RRBs, including verbal perseveration, hand flapping, body rocking, compulsive behaviors, and self-injurious behaviors, are commonly observed in FXS [8]; however, little is known about the trajectory of RRBs over time in individuals with FXS. The study’s findings revealed a peak in RRB severity between 7 and 12 years for all subscales, with the exception of sensory-motor behaviors, which peaked earlier before declining. Distinct RRB profiles in males and females with FXS emerged; however, significant overlap among the item and subscale levels of RRBs across gender was also observed. Importantly, a diagnosis of ASD significantly increased rates of RRBs across all subscale levels, and was identified as a major driver of sensory-motor behaviors in males with FXS when controlling for intellectual quotient (IQ) and age, as well as a likely driver of increased rates of restricted interests in individuals with FXS regardless of gender [8].

(4). Another study conducted by Cordeiro et al. focused on the quantification of social interaction skills in individuals with FXS. In this study, the Autism Screening Instrument for Educational Planning-Third Edition (ASIEP-3) was examined as a potential direct measure of social interaction skills in individuals with FXS, and feasibility of its use as a clinical trial outcome measure was evaluated. Parent-report questionnaires are currently the most commonly used measures of social development and ASD symptomatology in FXS research; however, they are limited by their subjective scores. The ADOS-2′s utility as an outcome measure in FXS research is significantly hindered by its inability to capture change in symptom severity over time. In this pilot study, the authors showed that the ASIEP-3 Interaction Assessment can be reliably administered across a wide range of ages and ability levels among males and females with FXS [9]. The score profiles correlated well with expected behaviors observed in FXS with ASD revealing a lower percentage of time engaged in interactions and more instances of decreased social responses without significant floor or ceiling effects. It also correlated well with other standard measures of social interaction. Additionally, the ASIEP-3 Interaction Assessment was shown to be patient-friendly with use of toys and objects of interest to each specific patient, as well as a time-efficient measure. Together, results from this study showed that the ASIEP-Interaction Assessment may be a promising and useful measure for the quantification of social interaction skills in individuals with FXS [9].

(5a and 5b). Two language-related articles by Shaffer et al. and Hoffmann et al., respectively, examined the feasibility and validity of various widely used standardized “normed” speech and language (SL) measures in FXS. While SL delay is a core issue in individuals with FXS, a difficulty inherent to assess it led to inconsistent data, and no consensus regarding the most reliable measure of SL in FXS, including in clinical trials. Namely, the norm-referenced assessments are incapable of providing a valid or scaled score for this population due to lack of sufficient lower-level items and resultant floor effect. Indeed, Hoffman et al. confirmed it for the Clinical Evaluation of Language Fundamentals-5th Edition (CELF-5) in particular, more so than either the Preschool 2nd Edition (CELF-P2) or the Bayley Scales of Infant and Toddler Development-III Edition (BSID-III), especially among older adolescents and young adults with FXS. The floor effects occurred less frequently among younger patients with FXS assessed by the CELF-P2 and BSID-III. Additionally, application of the CELF-P2 for older individuals who were unable to achieve a scaled score on the more age-appropriate CELF-5 was limited given the structure of the assessment and the need for high raw scores to earn an age-equivalent score. Despite these limitations, caregiver report tended to provide lower estimates of language ability than what was measured using the normed objective assessments [10]. (5b). The Expressive Language Sampling Narrative (ELS-N) has emerged as a promising new measure demonstrating its usefulness in a wide range of ages in developmental disabilities such as FXS and Down Syndrome and typically developing (TD) controls peers. To satisfy a high priority need to develop an appropriate SL outcome measure in FXS, Shaffer et al. also examined the ELS-N. They showed that individuals with FXS scored significantly differently than age-matched TD controls on all variables. While unintelligibility of speech, syntactic complexity, and lexical diversity provided the most pronounced differences, the impairments in these language areas were positively modulated by greater IQ and stronger adaptive skills. In contrast, ASD was related to less intelligibility in speech in females with FXS, and measures of hyperactivity were related to increased talkativeness and unintelligibility in all individuals with FXS [6]. Together, the data from this study revealed that the ELS-N is feasible to administer in individuals with FXS across a wide range of functioning and appears capable of capturing unique sex differences of the FXS phenotype, which suggests potential utility as a biomarker in future clinical trials in FXS.

(6). To better understand the health-related quality of life (HRQoL) in FXS, Coffman and colleagues examined the nature and degree of association between it and established measures of neurobehavioral functioning in FXS. Quality of Life (QoL) is an important outcome measure that can be utilized to validate a particular therapeutic intervention. Within the construct of QoL, HRQoL reflects an individual’s or group’s perception of how an illness or treatment affects their physical and neurobehavioral well-being over time. Results from this study revealed overall higher HRQoL ratings among parents of children with FXS who brought their children into a Fragile X Clinic compared to ones who completed HRQoL surveys online. Differences were attributed to a selection bias (i.e., higher functioning) and perceived parental competence (i.e., feel more comfortable bringing their child) of the in-person visit, and age (i.e., increasing age associated with improving HRQoL). Additionally, results from this study support previous findings demonstrating a consistent pattern of HRQoL concerns for parents of children with FXS with the highest HRQoL in physical and emotional functioning and lowest HRQoL in social functioning, as well as significant associations between HRQoL and measures of adaptive, maladaptive, and social behaviors [11].

(7). Luu and colleagues conducted the first meta-analysis examining the response to placebo in randomized controlled trials (RCTs) in FXS. Placebo response has steadily increased over time in neuropsychiatric clinical trials over time; however, the extent to which the placebo effect has affected FXS trial outcomes is unknown. This meta-analysis showed that patients with FXS receiving placebo can demonstrate statistically significant and clinically relevant placebo-related improvements on *caregiver*-rated but not on *clinician*-rated efficacy endpoints or *performance*-rated measures, although Clinical Global Impression Scale—Improvement ratings trended towards significance for placebo-related effects. Since commonly used outcome measures in the RCTs in FXS all showed statistically significant placebo response, including the Visual Analog Scale, Vineland-II, and ABC-CFXS, improvements in the study design and their implementations are necessary [12]. Assessment methods described in this Special Issue, which are in line with recommendations experts in the field of FXS [1] will contribute to that effort in future clinical trials in FXS.

(8). Scott and colleagues conducted further analyses of a prior RCT Cogmed data in FXS to better characterize those training data, identify predictors of training efficacy, and identify potential personal and/or environmental factors affecting clinical outcome in FXS. Improvement in working memory (WM) and executive functioning (EF) after completion of a computer-based WM training program (CogMed) has been previously demonstrated in FXS; however, factors affecting outcome are unknown. The pediatric participants of average IQ of 64 were divided in Cogmed Adaptive and Nonadaptive groups. Results of this “deeper dive” revealed that children with FXS who progress and expand memory capacity over time have better outcomes and perhaps a better response to intervention than those who show no improvement over time, even when adjusting for baseline cognitive ability. IQ and mental age, however, were related to clinical outcomes with higher mental age and IQ being linked to greater gains in WM. These analyses support a recommendation to include not only baseline feasibility but also a short-term capacity for training progress prior to inclusion and randomization, which may serve to better identify ones who are more likely to benefit from cognitive training across contexts. This further ensures that included participates are capable of not only performing the necessary cognitive tasks but also demonstrate the capacity for improvement over time [13].

In conclusion, we anticipate that the wide range of data published in this Special Issue will result in new and/or improved strategies that can be applied in FXS clinical trials and potential reduction in heterogeneity issues across FXS cohorts. The published data aim to address the gaps and limitations in knowledge related to objective outcome measures for successful clinical trials in FXS [1,2]. Despite tangible progress overall [2], translating the preclinical successes of the targeted interventions into clinical trials of humans in FXS still remains a challenge, and still no regulatory agencies have approved treatment in FXS. There should be a continued interest to expand on pilot data studies of valuable advanced imaging tracers to objectively measure receptor expression as a proxy of target engagement in FXS, and association with the FXS neurobehavioral phenotype. Together, the community effort needs to continue to generate reliable data on objective preferably biomarkers capacity measures for use in future clinical trials in FXS with and without ASD.
